# Supplementary material for: Establishment of a Screening Method for Epstein-Barr Virus-Associated Gastric Carcinoma by Droplet Digital PCR
Source: Microorganisms. 2019 Nov 29;7(12):628. doi: 10.3390/microorganisms7120628 (PMC6956032; doi:10.3390/microorganisms7120628)
Supplement: Supplementary file 1 [file microorganisms-07-00628-s001.pdf]

Tables

Table S1. Characteristics of gastric cancer from biopsy samples for evaluation of EBV-DNA load

Total	EBVaGC<br>n=21	Cotrol<br>n=5
Age, years, mean(range)	66.3 (42-85)	74.4(66-81)
Sex		
Male	20	3
Female	1	2
Histologic type		
Differentiated type	10	2
Undifferentiated type	11	3
Depth of tumor invasion		
Early stage	10	0
Advanced stage	11	5

EBVaGC Epstein-Barr virus-associated gastric carcinoma

Table S2 Characteristics of gastric cancer from serum samples for evaluation of EBV-DNA load

Total	EBVaGC<br>n=25	Control<br>n=24
Age, years, mean (range)	65.1 (36–91)	65.9 (37–87)
Sex		
Male	22	20
Female	3	4
Histologic type		
Differentiated type	10	10
Undifferentiated type	15	14
Depth of tumor invasion		
Early stage	16	14
Advanced stage	9	10

EBVaGC Epstein-Barr virus-associated gastric carcinoma
